# Exogenous Fatty Acids Modulate ER Lipid Composition and Metabolism in Breast Cancer Cells

**DOI:** 10.3390/cells10010175

**Published:** 2021-01-16

**Authors:** Angela Maria Rizzo, Irma Colombo, Gigliola Montorfano, Stefania Zava, Paola Antonia Corsetto

**Affiliations:** Department of Pharmacological and Biomolecular Sciences, Università degli studi di Milano, 20122 Milano, Italy; irma.colombo@unimi.it (I.C.); gigliola.montorfano@unimi.it (G.M.); stefania.zava@unimi.it (S.Z.)

**Keywords:** lipid metabolism, cancer, ER, fatty acids

## Abstract

(1) Background: Lipid metabolism is a fundamental hallmark of all tumors, especially of breast cancer. Few studies describe the different lipid metabolisms and sensitivities to the microenvironment of breast cancer cell subtypes that influence the proliferation, aggressiveness, and success of therapy. This study describes the impact of lipid microenvironment on endoplasmic reticulum (ER) membrane and metabolic activity in two breast cancer cell lines with Luminal A and triple-negative breast cancer (TNBC) features. (2) Methods: We investigated the peculiar lipid phenotype of a TNBC cell line, MDA-MB-231, and a Luminal A cell line, MCF7, and their different sensitivity to exogenous fatty acids (i.e., palmitic acid (PA) and docosahexaenoic acid (DHA)). Moreover, we verified the impact of exogenous fatty acids on ER lipid composition. (3) Results: The data obtained demonstrate that MDA-MB-231 cells are more sensitive to the lipid microenvironment and that both PA and DHA are able to remodel their ER membranes with consequences on resident enzyme activity. On the contrary, MCF7 cells are less sensitive to PA, whereas they incorporate DHA, although less efficiently than MDA-MB-231 cells. (4) Conclusions: This study sustains the importance of lipid metabolism as an innovative hallmark to discriminate breast cancer subclasses and to develop personalized and innovative pharmacological strategies. The different sensitivities to the lipid environment shown by MCF7 and MDA-MB-231 cells might be related to cell malignancy and chemoresistance onset. In the future, this new approach could lead to a substantial decrease both in deleterious side effects for the patients and in the cost of entire therapeutic treatments coupled with increased therapy efficiency.

## 1. Introduction

Cancer is a highly complicated disease because of its genetic and metabolic heterogeneity and complexity. In each cancer subtype, distinct genetic alterations, oncogenic signaling, and epigenetic changes are responsible for tumorigenesis [1]. Metabolic alterations represent a hallmark of cancer controlling tumor progression and metastasis [2]. The most understood metabolic perturbation in cancer cells is the “Warburg effect”, an energetically wasteful alteration of glucose metabolism [3]. Nevertheless, many altered metabolic pathways characterize cancer proliferation and are correlated with different extents to the cancer type and aggressiveness. While carbohydrate, protein, and amino acid metabolism in tumor cells has already been extensively dissected, lipid metabolism has only recently come to the attention of the scientific community. Although this field is still largely unexplored, great benefits could come from a deeper understanding of cancer cell lipid phenotype and the susceptibility to the lipid microenvironment.

In cells, lipids are responsible for maintaining cellular structures and for providing energy. Moreover, they are involved in cell signaling as precursors of biological active mediators. The bioactive lipid molecules are produced by the activation of multiple signaling pathways and, in cancer, might regulate multiple cellular effects [4,5,6]. Fatty acids (FA), glycerophospholipids (PL), sphingolipids (SL), and sterols, in particular, cholesterol (Chol), have relevance in cancer development and chemotherapy response. For instance, FA are needed for energy storage, membrane structures, and precursors of signaling molecules and SL are not only structural components of cell membranes but also bioactive lipid molecules involved in apoptosis and/or chemoresistance [7]. In order to build more complex lipids, FA are derived from either an exogenous source, such as diet, or from de novo synthesis. Indeed, most of the enzymes and carriers involved in lipid synthesis and uptake are abnormally expressed in cancer cells, and their chemical and genetic inhibition might reduce proliferation or induce apoptosis [8]. Concerning the exogenous source, previous studies have indirectly suggested that cancer cells utilize mainly exogenous FA for energy or membrane synthesis. For example, Nieman et al., by co-culturing ovarian cancer cells and adipocytes, demonstrated that cancer cells, for their metabolic needs, obtain lipids from neighboring adipocyte stores [9]. Once in the active cell pool, FA might also contribute to the constitution of structural lipids, such as SL, PL, and Chol, and non-structural lipids, such as triglycerides (TG) and cholesteryl esters (CE), that might be stored in cellular lipid droplets.

The characterization of many metabolic pathways sustained by cancer cells to retrieve nutrients for growth and proliferation indicates that cancer metabolism is highly heterogeneous and subject to external cues. These observations have great impact on chemotherapy and immunotherapy efficacy [10].

Breast cancer is the most commonly diagnosed cancer and one of the leading causes of cancer death among women worldwide (American Cancer Society, 2015). Indeed, the biological heterogeneity of this tumor, due to different molecular subtypes, risk factors, clinical behavior, and responses to treatment, represents the major obstacle to therapy success and survival. In particular, studies based on global gene expression analyses have identified four main molecular subtypes of breast cancer known as Luminal A, Luminal B, HER2-enriched (HER2E), and Basal-like. In addition to these subtypes, breast cancer negative for an estrogen receptor, a progesterone receptor, and HER-2 is defined as a triple-negative breast cancer (TNBC). All these breast cancer subtypes have been also characterized based on their significant differences in terms of incidence, risk factors, baseline prognosis, age at diagnosis, and response to treatment [11].

Advances in treatment strategies, including surgery, radiotherapy, and chemotherapy, have greatly increased the overall survival rate, but it is still challenging to select a personalized therapeutic strategy for the treatment of progressive and advanced breast cancers.

The plasma lipid profile in breast cancer patients has been extensively investigated and alterations have been associated with cancer development. However, the prognostic value of serum lipid markers in cancer and the beneficial roles of nutritional interventions are still under debate [12]. Few studies describe the different lipid metabolisms and sensitivities to lipid environment of breast cancer cell subtypes that might determine breast cancer proliferation, aggressiveness, and response to chemotherapy. Therefore, in this study, we investigate both the peculiar lipid phenotype of a TNBC cell line, MDA-MB-231, and a Luminal A cell line, MCF7, and their sensitivity to exogenous FA (i.e., palmitic and docosahexaenoic acids) in order to evaluate the impact of a lipid microenvironment on tumor metabolism.

## 2. Materials and Methods

### 2.1. Materials

Palmitic acid (PA, sodium salt) and cis-4,7,10,13,16,19-docosahexaenoic acid (DHA, sodium salt) were purchased from Sigma-Aldrich (St. Louis, MO, USA). Palmitic acid was dissolved in 50% ethanol, while docosahexaenoic acid was dissolved in 100% ethanol to a concentration of 10 mg/mL; these stock solutions were stored at −80 °C under N_2_ until use. The rabbit monoclonal anti-Calnexin (C5C9) and the rabbit monoclonal anti-Histone H3 (D1H2) antibodies were purchased from Cell Signaling Technology, Danvers, MA, USA. The mouse monoclonal anti-HSP60 (66041) antibody was purchased from Proteintech, Deansgate, Manchester, UK. Bound primary antibody was visualized by proper secondary horseradish peroxidase (HRP)-linked antibody, purchased from Cell Signaling Technology, Danvers, MA, USA. All solvents were purchased from Carlo Erba Reagents, Italy. Avanti Polar Lipids Inc. (Alabaster, AL, USA) supplied the fatty acid methyl ester (FAME) standards for GC analysis and neutral lipids (TG and CE) for LC analysis. Phospholipid standards were purchased from Sigma Aldrich, St. Louis, MO, USA.

### 2.2. Cell Lines and Fatty Acid Treatment

Human breast cancer cells MDA-MB-231 (Basal-like and TNBC) and MCF7 (Luminal A) were provided by ATCC. Both cell lines were routinely maintained in Dulbecco’s Modified Eagle’s Medium (DMEM) supplemented with 10% fetal bovine serum (FBS), 100 U/mL penicillin, 100 mg/mL streptomycin, and 2 mM glutamine (Gibco-BRL, Life Technologies Italia srl, Monza, Italy). Cells were grown at 37 °C in 5% CO_2_ at 98% relative humidity. Before treatments, 1.5 × 10^4^ cells/cm^2^ of MDA-MB-231 cells and 3 × 10^4^ cells/cm^2^ of MCF7 cells were seeded to adhere with 18 mL of DMEM containing 10% *v*/*v* FBS for 48 h. After 48 h, the medium was replaced with fresh medium supplemented with PA or DHA. To this aim, the FA stock solutions were diluted in culture medium at 50 µM and supplemented with fatty acid free bovine serum albumin (BSA) to a final molar ratio of 6:1 for PA and of 5:1 for DHA. Both cell lines were treated for 72 h with FA. The experiments included control cells (Ctr) treated with equal concentrations of ethanol, always less than 1%.

### 2.3. Endoplasmic Reticulum Isolation

The isolation of endoplasmic reticulum (ER) was performed using a commercial kit (ER0100, Sigma-Aldrich, St. Louis, MO, USA) following the manufacturer’s instructions with minor modifications. These modifications allowed for isolation of the microsomal fractions from pellets of 200 × 10^6^ MDA-MB-231 and MCF7 cells. The protocol allowed to purify sequentially the following cell fractions: PNS (post nuclear surnatant), P1 (nuclei, heavy mitochondria and membrane sheets), PMF (post mitochondrial fraction), P4 (mitochondria, lysosome, peroxisome, Golgi membranes, and endoplasmic reticulum), and finally CMF (microsomal fraction containing endoplasmic reticulum). The CMF was characterized as a fraction highly enriched with the ER cellular compartment by western blot, as described later.

In details, the PNS was centrifuged at 12,000× *g*, instead of 10,000× *g*, to allow for better separation of the mitochondrial fraction or supernatant (PMF) to the pellet (P4). Then, the pellet (P4) was resuspended in an IBc buffer (100 μL Tris-MOPS 0.1 M + 10 μL EGTA/Tris 0.1 M + 100 μL saccharose 1 M + 1 μL of protease inhibitor cocktail 10 × + 689 μL H_2_O ultrapure) and centrifuged at 7000× *g* for 10 min at 4 °C. The resulting pellet (CMF), enriched in ER, was resuspended with 100 μL of isotonic extraction buffer 1 × (10 mM HEPES (4-(2-hydroxyethyl)-1-piperazineethanesulfonic acid), pH 7.8, with 1 mM EGTA, and 25 mM potassium chloride) and stored at −20 °C for further analyses.

The protein content of each fraction was determined by the Lowry assay [13].

### 2.4. Lipid Extraction and Analysis

Whole cell pellets and CMF fractions were extracted with three different chloroform/methanol mixtures 1:1, 1:2, and 2:1 (*v*/*v*) and partitioned with chloroform/methanol/water, 47:48:1, *v*/*v*/*v* and then with water. Each solvent contained 50 μM 2,6-bis(1,1-dimethylethyl)-4-methylphenol (BHT) to protect lipids from oxidation. The organic phase was dried and resuspended in chloroform/methanol (2:1) for the analysis of total FA, PL, and neutral lipids (TG and CE) [14].

Total FA were determined as methyl esters (FAME) by gas chromatography. The methyl esters were obtained by derivatization with 3.33% (w/v) sodium methoxide in methanol and injected into an Agilent Technologies (6850 series II) gas chromatograph equipped with a flame ionization detector and a capillary column (AT Silar) (length 30 m, film thickness 0.25 μm). The carrier gas was helium, the injector temperature was 250 °C, the detector temperature was 275 °C, and the oven temperature was set at 50 °C for 20 min and then increased to 200 °C at 10 °C min^−1^ for 20 min. A standard mixture containing all FAME was injected for calibration, and TG C17:0 was added before sample manipulation and used as internal standard [15].

Specific fatty acid ratios were utilized to calculate the relative activity of ER key enzymes of lipid metabolism: desaturases Δ5D (20:4n-6/20:3n-6), Δ6D (18:3n-6/18:2n-6), stearoyl-CoA desaturase 1 (SCD-1 n-7: 16:1/16:0, SCD-1 n-9: 18:1/18:0), elongases Elovl-6 (18:0/16:0), Elovl5 (20:3 n-6/18:3 n-6), and de novo lipogenesis or DNL (16:0/18:2n-6).

The PL and neutral lipid (NL) quantifications were achieved as previously described by high-pressure liquid chromatography (HPLC) (Jasco, Tokyo, Japan) equipped with an ELSD detector (Sedere, Alfortville Cedex, Paris, France) and silica normal-phase LiChrospher Si 60 column (LiChroCART 250-4; Merck, Darmstadt, Germany) [16].

### 2.5. Western Blot

To confirm the purity grade of microsomal fractions, the presence of both non-ER biomarkers (Heat Shock Protein HSP60 and Histone H3) and an ER biomarker (Calnexin) was determined in the CMF fraction of each sample by western blot.

Ten micrograms of the protein from each CMF fraction, purified from control and treated cells, were separated on 10% SDS-PAGE gel and transferred to a polyvinylidenedifluoride transfer membrane (PVDF) (Bio-Rad). Membranes were then probed with the following antibodies: rabbit anti-Calnexin monoclonal antibody (1:1000) marker of endoplasmic reticulum, rabbit anti-Histone H3 monoclonal antibody (1:2000) nucleus marker, and anti-mouse anti-HSP60 monoclonal antibody (1:400) mitochondria marker.

The blots were incubated for 1 h at room temperature with horseradish peroxidase-linked to the appropriate secondary antibodies diluted 1:3000 in a blocking buffer. All blots were developed by the ECL Western Blotting Detection LiteAblot^®^ plus Kit Reagent (Euroclone S.P.A, Pero, Italy) following the manufacturer’s protocol.

The immunocomplexes were detected by the Li-Cor Odyssey FC system (LI-COR^®^ Biosciences, Lincoln, NE, USA) and the relative intensities of the chemiluminescent signals were quantified by a digital scanner.

### 2.6. Immunofluorescence

For immunofluorescence analyses, MCF7 and MDA-MB-231 cells were cultured on 8-well slides. After 72 h of treatment, cells were fixed with 4% paraformaldehyde in phosphate-buffered saline (PBS) at room temperature for 10 min; permeabilized in ice-cold 0.5% Triton X100, HEPES buffer (4-(2-hydroxyethyl)-1-piperazineethanesulfonic acid) for 4 min; and blocked with 1% bovine serum albumin (BSA) in PBS for 10 min.

For the detection of the ER, rabbit monoclonal anti-Calnexin antibody (1:100) followed by incubation with goat anti-rabbit IgG-Alexa Fluor 488 (1:1000) was used.

Actin filaments were stained by the incubation of cells in Phalloidin (Phalloidin Tetramethylrhodamine B isothiocyanate, Sigma-Aldrich; St. Louis, MO, USA, 50 ug/mL). Lipid droplets were stained by incubation with Bodipy ((E,E)-3,5-bis (4-phenyl-1,3-butadienyl)-4,4-difluoro-4-bora-3a,4a-diaza-s-indacene) (493/503, Molecular Probe, Invitrogen, Life Technologies, Carlsbad, CA, USA) (0.001 mg/mL).

The nuclei of cells were stained with 4′,6-diamidino-2-phenylindole (DAPI; Sigma-Aldrich, St. Louis, MO, USA) (0.01 mg/mL).

The fluorescence images were acquired using an Eclipse TE200 inverted microscope equipped with immersion objective at 60× magnification and digital camera (Nikon, Tokyo, Japan).

### 2.7. Statistical Analysis

All data are expressed as mean ± SE. Student’s unpaired *t*-test was utilized for comparisons between the two cell lines and to compare treated and control cells. The level of statistical significance was set at *p* < 0.05.

## 3. Results

### 3.1. Impact of Lipid Microenviroment on Lipid Phenotype of MCF7 and MDA-MB-231 Breast Cancer Cells

#### 3.1.1. Effect of Exogenous Fatty Acids on Cell Lipid Profile

To evaluate the different effects of exogenous FA, we chose two kinds of breast cancer cell lines, MDA-MB-231 and MCF7, characterized by several phenotypic and genotypic differences [17]. The MCF7 cell line is characterized by positive estrogen receptor and/or progesterone receptor expression and exhibits a high level of luminal feature-correlated genes/proteins such as ERα or luminal keratins and transcription factors such as GATA3 and FOXA1. These cells, classified as Luminal A, are more differentiated and have a poor ability to migrate due to high cell–cell junctions. On the contrary, the MDA-MB-231 cell line is characterized by low or no expression of all three receptor markers and by high invasivity and aggressiveness; this line is classified as triple negative breast cancer (TNBC). In addition, in relation to the metabolic features, MCF7 cells are more Pasteur type, relying on ATP production from oxidative phosphorylation at normoxic conditions but increasing their glycolytic activity under hypoxia, while MDA-MB-231 cells are more Warburg type, mainly relying on glycolysis for ATP production under both normoxic and hypoxic conditions [18].

Few data are available about their specific lipid metabolic phenotype and their sensitivity to microenvironment modulation. As little information is also available in relation to the sensitivity of these cell lines to exogenous lipids, we preliminary exposed MDA-MB-231 and MCF7 cells to increasing concentrations of a saturated fatty acid (PA) and an unsaturated fatty acid (DHA) for 72 h. Palmitic acid is the most common saturated fatty acid in the human diet, and there is growing evidence that highlights its specific tumorigenic properties and its ability to increase the metastatic features of cancer cells [19]. Among unsaturated FA, in vitro and in vivo studies demonstrate that omega-3 PUFA, especially DHA, enhance the sensitivity of cancer cells to chemotherapy. Indeed, DHA incorporation induces the alteration of gene expression, modulation of cellular proliferation, and differentiation, increasing drug transport across the cell membrane and generation of reactive oxygen species (ROS) [20].

The cell viability and apoptotic effect were measured by the MTT test and cytofluorometry, respectively (Figures S1 and S2). As shown in Figure S1, PA induces a progressive reduction of cell viability in MDA-MB-231 cells; in particular, while the viability is significantly reduced at PA concentrations above 100 µM, the exposure to 50 µM PA does not appear to be cytotoxic. On the contrary, the MCF7 cell line does not show significant changes in viability at any PA concentrations. Considering DHA treatments, we observe a significant reduction in MDA-MB-231 cell viability from 200 µM DHA while no effects are revealed for MCF7 (Figure S1). Collectively, these results suggest that the MCF7 cell line is less sensitive to treatment with both exogenous FA compared to MDA-MB-231 cells. Based on the MTT assays, PA and DHA were used at 50 µM concentration in subsequent experiments.

To further investigate the possible toxic effects of exogenous FA, we measured apoptosis by Annexin V assay; the results confirm the minor responsiveness of MCF7 with respect to MDA-MB-231 cells (Figure S2). Indeed, treatment with both exogenous FA at 50 µM slightly, even if significantly, reduces the viability only of MDA-MB-231 cells and significantly increases the number of cells in both early and late apoptosis to a total value of 20%. On the other hand, the MCF7 cell line also shows a slight but significant reduction of viable cells after treatment with 50 µM PA or DHA but without indication of apoptosis.

Having established that the exposure of both cell lines to 50 µM PA or DHA does not cause severe cytotoxicity, next, we evaluated the different modulations induced by saturated FA, as PA, and unsaturated FA, as DHA, on lipid phenotype in MCF7 and MDA-MB-231 cells.

As shown in Table S1, the evaluation of total fatty acid content and composition has highlighted that both cell lines show, at baseline, a different and peculiar phenotype. In particular, MCF7 cells have a significant higher content of monounsaturated fatty acids (MUFA) than MDA-MB-231 (40.5 ± 0.665 vs. 33.5 ± 0.214), and a significant lower content of polyunsaturated fatty acids (PUFA) (21.4 ± 0.190 vs. 27.2 ± 0.303). The MCF7 cells have a lower content of both n-6 (15.2 ± 0.297 vs. 17.3 ± 0.229) and n-3 PUFA (6.21 ± 0.213 vs. 9.89 ± 0.212). This different fatty acid profile detected in the analyzed cell lines suggest a peculiar lipid metabolism that might be responsible for different susceptibilities to lipid microenvironment changes simulated by PA and DHA treatments. Indeed, treatment with 50 µM PA for 72 h determines the significant changes in lipid phenotype in MDA-MB-231 but not in MCF7 cells. Specifically, PA exposition significantly increases the saturated fatty acid (SFA) percentage, both palmitic acid and stearic acid (C18:0), and decreases MUFA, especially oleic acid (C18:1, from 30.0 ± 0.226 to 24.1 ± 0.615), and PUFA, especially arachidonic acid (C20:4, from 10.6 ± 0.170 to 9.03 ± 0.337) and docosahexaenoic acid (C22:6, from 4.30 ± 0.077 to 3.82 ± 0.071) (Table S1). On the contrary, both cell lines exhibit significant modulation of the FA profile after DHA treatment (Table S1).

**Figure 1:**
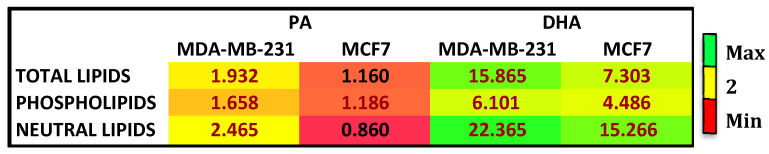
Enrichment heat map of palmitic acid (PA) and docosahexaenoic acid (DHA) in total lipids, phospholipids, and neutral lipids isolated from breast cancer MDA-MB-231 and MCF7 cells after treatment with PA or DHA (50 µM) for 72 h expressed as fold changes over control cells.

To further characterize the effects of treatments and the cell fatty acid fate, we analyzed the fatty acid composition of complex lipids, such as phospholipids (PL) and neutral lipids (TG and CE). Figure 1 reports the enrichment in each cell line after PA and DHA treatment relative to control, untreated cells. In total cell lipids after treatment with exogenous FA, DHA was more enriched in comparison to PA in both cell lines; among complex lipids, PA and DHA were highly enriched in neutral lipids (NL), more so than PL. Altogether, the results collected highlight that, at 72 h after treatment with PA or DHA, the TNBC cell line, MDA-MB-231, has a higher incorporation and metabolism to PL and NL of exogenous FA compared to MCF7 cells.

**Figure 2:**
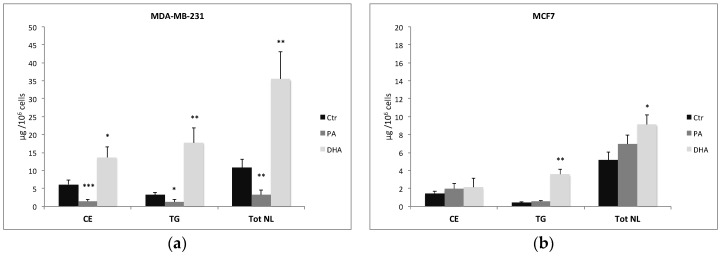
Content (μg/10^6^ cells) of total neutral lipids (NL), cholesteryl esters (CE), and triglycerides (TG) isolated from breast cancer cells MDA-MB-231 (**a**) and MCF7 (**b**) treated with PA or DHA (50 μM) for 72 h: data are represented as mean ± SE obtained from four independent experiments. (* *p* < 0.05, ** *p* < 0.01, and *** *p* < 0.001 vs. Ctr cells of the same line).

The characterization and quantification of NL isolated from control and treated cells show not only a change in FA composition but also a significant alteration of their concentration, especially in MDA-MB-231 cells (Figure 2a). As a matter of fact, PA treatment and incorporation determine a significant reduction of CE and TG in MDA-MB-231 cells but not in MCF7 cells. On the contrary, DHA incorporation and metabolism determine a significant increase of both CE and TG in MDA-MB-231 cells but only of TG in MCF7 cells (Figure 2b).

#### 3.1.2. Effect of Exogenous Fatty Acids on Lipid Droplets

**Figure 3:**
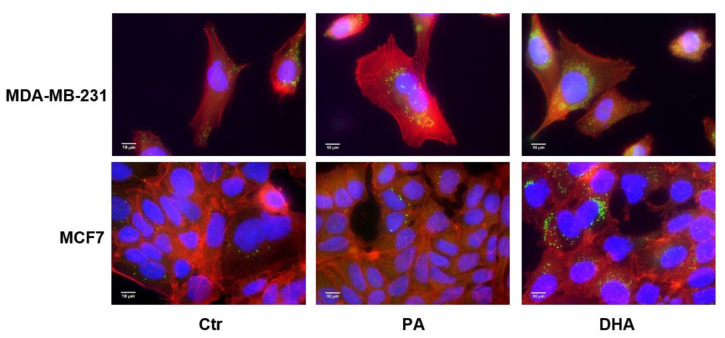
Immunofluorescence analysis of lipid droplets in estrogen-sensitive breast cancer cells MCF7 (Luminal A) and estrogen-insensitive MDA-MB-231 (basal-Like and triple-negative breast cancer (TNBC)) following treatment with PA or DHA (50 μM) for 72 h: 4′,6-diamidino-2-phenylindole (DAPI) (nucleus in blue), Bodipy (lipid droplet in green), and phalloidin (cytoskeleton in red).

Due to modulation of the NL content, we decided to analyze the cellular distribution of lipid droplets as the apolar core of these structures is constituted by TG and CE in addition to the enzymes that regulate their storage and hydrolysis [21]. As shown in Figure 3, after DHA incorporation and metabolism, the content of lipid droplets undergoes an increase in both cell lines.

Moreover, as can also be seen in Figure 3, after FA treatment, the lipid droplets seem to mainly localize adjacent to nuclear membranes, which are known to be associated with the endoplasmic reticulum (ER).

### 3.2. ER Membrane Remodelling Induced by Exogenous Fatty Acids

**Figure 4:**
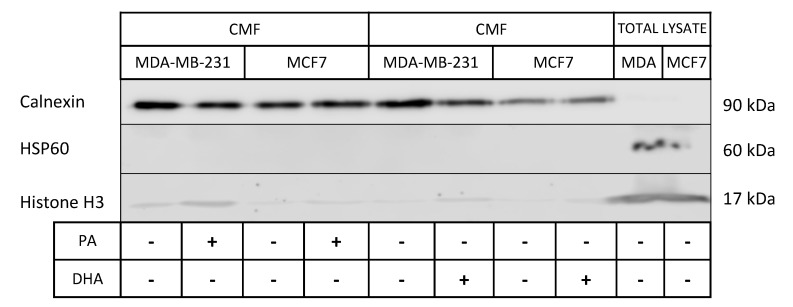
Characterization of microsomal fractions (CMF) purified from control and PA- or DHA-treated breast cancer MDA-MB-231 and MCF7 cells (50 µM) for 72 h: the purity of the microsomal fractions (endoplasmic reticulum (ER)) is evaluated using positive ER markers, such as Calnexin, and markers from other cell compartments, such as HSP60 and histone H3.

The association between lipid droplets and the ER is strictly correlated with hydrophobic lipid ester transport, which drives lipid trafficking, important for membrane structure and energy storage [22]. As the strong ER involvement in lipid droplet synthesis and lipid metabolism is also well known, we analyzed the impact of exogenous saturated and unsaturated fatty acids (PA and DHA) on ER lipid composition and metabolic activity. To this aim, we purified microsomal fractions enriched in ER from MCF7 and MDA-MB-231 cells. The characterization of ER fractions, isolated as CMF from the control and PA- or DHA-treated cells, with specific protein markers is reported in Figure 4; the isolated ER fractions show an enrichment of the ER protein biomarker, calnexin, and a very low level of mitochondrial and nuclear protein biomarkers HSP60 and Histone H3, respectively.

**Figure 5:**
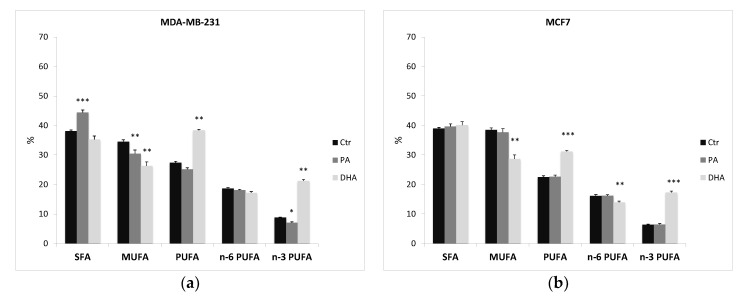
Percentage distribution of fatty acids from ER fractions purified from MDA-MB-231 (**a**) and MCF7 cells (**b**), treated with PA and DHA (50 µM) for 72 h: data are represented as mean ± SE of four independent experiments. (* *p* < 0.05, ** *p* < 0.01, and *** *p* < 0.001 vs. Ctr cells of the same line). SFA, saturated fatty acids; MUFA, monounsaturated fatty acids; PUFA, polyunsaturated fatty acids (=sum of n-6 and n-3 PUFA); n-6 PUFA, omega-6 polyunsaturated fatty acids; and n-3 PUFA, omega-3 polyunsaturated fatty acids.

After ER purification, we also extracted and analyzed the microsomal lipids in control and treated cells. Table S2 and Figure 5 indicate that the fatty acid profile is consistent throughout the cell structures and that, once again, a different fatty acid profile between the two cell lines is evident. Indeed, also ER fractions from MCF7 cells have a higher content of MUFA than MDA-MB-231 cells (38.5 ± 0.662 vs. 34.5 ± 0.768), especially of palmitoleic acid (C16:1; 10.1 ± 0.448 vs. 4.87 ± 0.588), and a lower level of PUFA (22.5 ± 0.450 vs. 27.4 ± 0.405), especially of n-3 PUFA (6.37 ± 0.138 vs. 8.79 ± 0.455).

Furthermore, the PA treatment determines a significant alteration in lipid composition of the ER in MDA-MB-231 cells. Indeed, PA incorporation induces a significant increase of SFA (from 38.1 ± 0.765 to 44.4 ± 0.595) and a significant reduction of MUFA (from 34.5 ± 0.768 to 30.5 ± 0.884), especially of oleic acid (from 29.6 ± 0.576 to 26.0 ± 1.15). On the contrary, PA does not affect the ER lipid profile in MCF7 cells.

Interestingly, n-3 fatty acid DHA incorporation dismantles both cell-line ER lipid assets, inducing a significant improvement of n-3 PUFA and a reduction of MUFA distribution. Noteworthily, DHA does not modify the SFA content and it seems to promote a metabolic retro conversion of DHA to eicosapentaenoic acid (EPA) (C20:5), especially in MCF7, with an increase from 1.55 ± 0.093 to 5.10 ± 0.181. The similar distribution detected in whole cells (Table S1) and ER fractions (Table S2) demonstrates that ER, a key site for lipid biosynthesis, is altered by exogenous FA.

**Figure 6:**
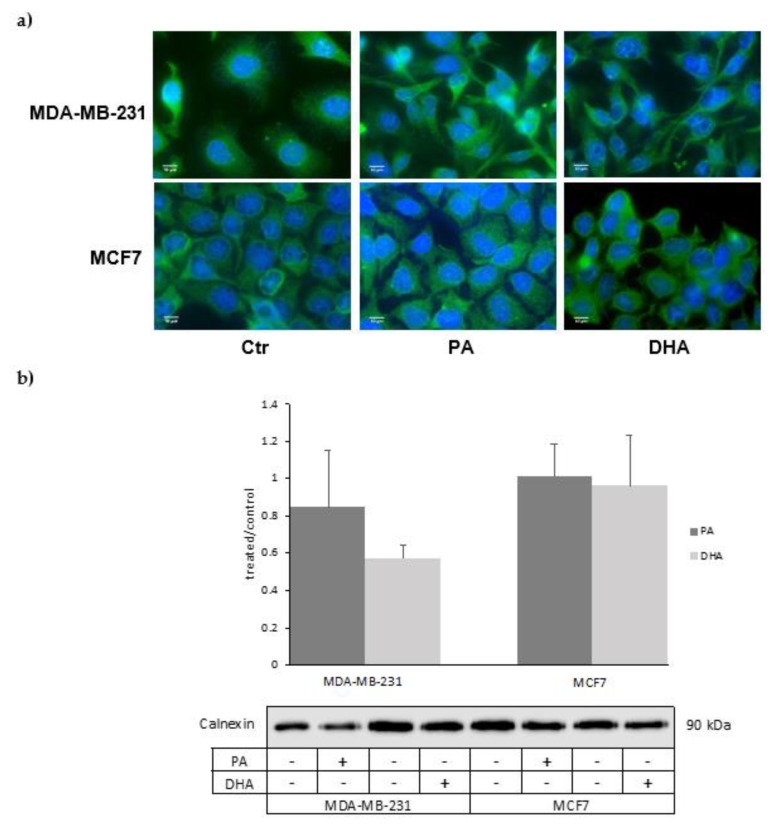
(**a**) Immunofluorescence analysis of the ER structure and (**b**) calnexin level in ER fractions of MDA-MB-231 and MCF7 cells treated with PA or DHA (50 µM) for 72 h: DAPI (nucleus in blue) and calnexin (ER in green).

In addition, preliminary data obtained by immunofluorescence analyses suggest that FA treatments might also induce ER membrane remodeling (Figure 6a). These structural changes in MDA-MB-231 cells treated with DHA are also suggested by the reduction, even if no statistically significant, of the ER marker calnexin analyzed by western blot (Figure 6b).

The ER has a crucial role in metabolic pathways that synthesize a variety of lipids, including FA, PL, Chol, and NL [23]. Many key enzymes involved in lipid synthesis are embedded in the ER membrane. For example, FA elongation and desaturation occur in the ER membrane [24,25]. The activity of the enzymes involved in lipid metabolism can be estimated using product-to-precursor fatty acid ratios. In particular, we calculated the relative activities of the delta 5 desaturase (Δ5D; 20:4n-6/20:3n-6), delta 6 desaturase (Δ6D; 18:3n-6/18:2n-6), stearoyl-CoA desaturase 1 (SCD1n-7; 16:1n-7/16:0, and SCD1 n-9; 18:1n-9/18:0), de novo lipogenesis or DNL (16:0/18:2n-6), elongase 6 (Elovl-6; 18:0/16:0), and elongase 5 (Elovl-5; 20:3 n-6/18:3 n-6) [26,27]. The ratios were calculated from the amount of FA isolated in ER fraction normalized by protein content (µg FA/mg proteins).

**Figure 7:**
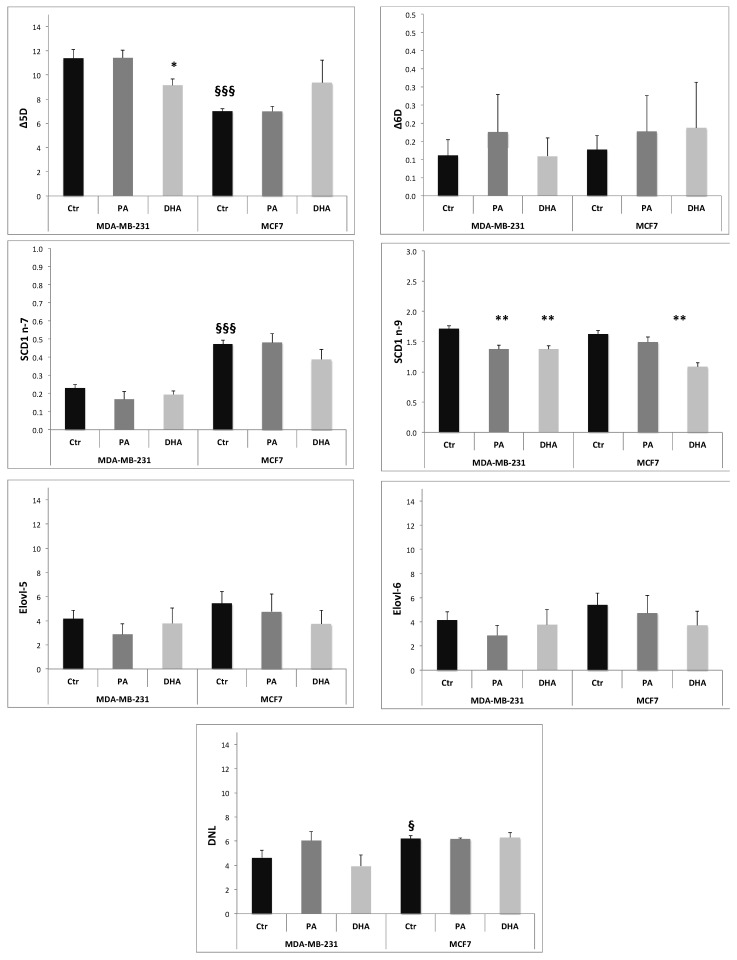
Activities of enzymes involved in fatty acid metabolism, estimated using product-to-precursor fatty acid ratios: The data are expressed as mean ± SE. * *p* < 0.05 and ** *p* < 0.01, vs. own Ctr; § *p* < 0.05 and §§§ *p* < 0.001 vs. MDA-MB-231 Ctr.

The data obtained, reported in Figure 7, suggest that, at baseline, the MCF7 cell line has a higher activity of SCD1 n-7 and de novo lipogenesis (DNL) and a lower activity of Δ5D compared to MDA-MB-231 cells. The PA incorporation induces a decrease of SCD-1 n-9 activity only in MDA-MB-231 cells, while DHA treatment determines a significant reduction of Δ5D in MDA-MB-231 cells and of SCD-1 n-9 in both cell lines. Noteworthily, both exogenous FA, PA and DHA, do not affect de novo lipogenesis, indicating that the measured TG increase induced by DHA exposure might be related to an improvement of lipid storage activity. Our data suggest that DNL in breast cancer cells is a key metabolism strongly resilient to microenvironment manipulations.

## 4. Discussion 

Molecular mechanisms of tumor progression and therapeutic strategies remain crucial subjects for investigation. Metabolic alterations are a hallmark of cancer controlling tumor progression and metastasis. The most understood metabolic perturbation in cancer cells is the “Warburg effect”, a phenomenon observed in a large variety of tumor types that consume high glucose amounts, even under aerobic conditions [28]. A crucial role in tumor progression has been also exerted by alterations of the tricarboxylic acid cycle and the metabolic pathways of serine, glycine, and glutamine [29,30].

Nevertheless, many studies have provided strong evidence for reprogramming of lipid metabolism in a tumor [31,32]. Different lipid synthesis and transport inhibitors have shown promising antitumor effects in preclinical and early phases of clinical trials [33]. However, there are again many troubles in developing personalized cancer strategies based on targeting altered lipid metabolism. These problems are closed to incomplete knowledge of the mechanisms that modulate lipid synthesis, storage, utilization, and efflux in cancer cells. Moreover, they could be also related to the specific lipid profiles and metabolism that characterize each tumor type and its peculiar sensitivity to the lipid microenvironment.

Some studies highlight the link among lipid metabolism, growth, and metastasis in breast cancer. For example, a recent report illustrated that 27-hydroxy cholesterol is synthesized from cholesterol within cancer cells and increases breast cancer growth and metastasis, binding to estrogen receptor alpha and activating oncogenic estrogen signaling [34]. The expression of cytochrome P450 CYP27A1, which converts cholesterol to 27-hydroxy cholesterol, is high in epithelial breast tumors, and its expression is positively associated with the tumor grade [35]. Moreover, the chemotherapy significantly alters plasma lipids and apolipoprotein levels in cancer patients, especially in breast cancer patients; indeed, many anticancer drugs target lipid metabolism [36]. The antiestrogen tamoxifen, for example, demonstrates several nongenomic activities, such as the inhibition of sphingolipid metabolism [37]. Chemotherapy drugs also affect 3-hydroxy-3-methylglutaryl coenzyme A reductase (HMGCoAR), the rate-limiting enzyme of cholesterol synthesis. Doxorubicin decreases HMGCoAR protein levels, while paclitaxel increases HMGCoAR expression; on the contrary, no lipid effect was associated with cyclophosphamide. These data suggest variable effects of chemotherapy drugs on cholesterol synthesis that might also be related to drug efficacy [38].

Unfortunately, the lipid mechanisms operating in different breast cancer types, such as the estrogen receptor negative tumor cells, are still lacking. Therefore, in order to ensure the success of chemotherapy and immunotherapy, the discovery of new connections between lipid environment and cancer lipid phenotype could not only shed light on carcinogenesis but also reveal new principles that establish and maintain the tumorigenic state and allow for the selection of a personalized therapeutic strategy.

With our study, we demonstrate that two different types of breast cancer cell lines exhibit specific sensitivity to lipid modulation. As a matter of fact, after 72 h of exogenous FA treatment, we observe a selective sensitivity in the TNBC cell line MDA-MB-231 compared to the Luminal A cell line MCF7 correlating with a distinct lipid phenotype. This specific cell response is strictly closed to the peculiar lipid profile and metabolism that characterizes each cell subset. In addition, we demonstrate that the long exposure to exogenous FA affects the lipid composition of the ER membrane. In particular, DHA treatment determines a significant alteration of the MUFA and PUFA contents of the ER compartment in MDA-MB-231 cells, with consequent changes in resident enzyme activity. Indeed, the ER membrane is the site where FA can be further modified through elongation and desaturation processes.

On the basis of different substrate specificity, elongases (Elovl) might be divided into four groups: the first group elongates SFA and MUFA (Elovl-1, Elovl-3, Elovl-6, and Elovl-7). The second group includes elongase 2 (Elovl-2) that elongates PUFA, while the third group, that is elongase 5 (Elovl-5), catalyzes the elongation of long chain PUFA (18–22 carbons). Finally, the last group is elongase 4 (Elovl-4) that elongates saturated as well as unsaturated very long chain FA.

Concerning oxidative desaturation, mammals express different acyl-CoA desaturases, which include mainly Δ5 desaturase (Δ5D), Δ6 desaturase (Δ6D), and Δ9 desaturase (Δ9D) (also known as stearoyl-CoA desaturase or SCD). Δ5D and Δ6D catalyze the biosynthesis of PUFA, and their most preferred substrates are FA with C16–C24, mainly of exogenous origin as linoleic acid and α-linolenic acid. Δ9D catalyzes the introduction of a double bond into SFA with the preferred substrates as PA (C16:0) and stearic acid (C18:0) [39].

A variety of enzymes associated with the ER membrane and involved in lipid metabolism are able to regulate the behavior of cancer cells. For instance, a high expression of fatty acid synthase and stearoyl CoA desaturase 1 (SCD1) is associated with relatively high risk of lung carcinoma and with poor patient prognosis [40,41].

Our study indicates that exogenous FA, especially PUFA, are able to perturb ER membrane architecture, modulating desaturase activity, in particular Δ5 desaturase and SCD1, especially in MDA-MB-231 cells.

Overall, our results do not suggest any alteration of de novo lipogenesis in either cell line after 72 h treatment. Indeed, in these cells, FA are mainly esterified, resulting in TG and CE synthesis followed by their storage in lipid droplets, ubiquitous ER-derived organelles. Lipid droplet synthesis is thought to occur through the TG accumulated between the leaflets of ER, which bud into the cytoplasm. Although in the past lipid droplets were considered inert fat depots, recently, many roles beyond energy storage are emerging, including regulation of lipid trafficking, protein management and quality control, ER homeostasis, and autophagy [42,43]. Of note, lipid droplets provide lipids, such as PUFA, that act as signaling molecules by interacting with transcription factors such as peroxisome proliferator-activated receptors (PPARs) and sterol-regulatory element binding proteins (SREBPs), or they are converted into bioactive lipid mediators, such as eicosanoids, that act as paracrine and autocrine messengers affecting inflammatory signaling, metabolism, proliferation, migration, and metastasis [44,45]. Then, we suggest that the increase in lipid droplets after DHA cancer cell exposition might be correlated to ER remodeling; this effect, particularly evident in the MDA-MB-231 cell line, might be correlated with the DHA-sensitizing effect in chemotherapy.

## 5. Conclusions

Lipid metabolic reprogramming and tumor microenvironment susceptibility are emerging mechanisms that significantly influence the success of chemotherapy and immunotherapy. Given that, a specific lipid phenotype of breast cancer cells might be responsible for resistance onset.

This study demonstrates not only that the analyzed cell lines with Luminal A and TNBC features have a different lipid phenotype and a peculiar lipid metabolism that influences their sensitivity to the microenvironment but also that it sustains the importance of lipid metabolism as an innovative hallmark to discriminate breast cancer subclasses and to develop personalized and innovative pharmacological strategies.

## Data Availability

Data presented in this study are contained within this article and the supplementary materials or available upon request to the corresponding authors.

## Supplementary Materials

The following are available online at https://www.mdpi.com/2073-4409/10/1/175/s1, Figure S1: Effects of PA and DHA on cell viability of MDA-MB-231 and MCF7 cells analyzed by MTT assay, Figure S2: Analysis of the apoptotic process by Annexin V cytofluorimetry in MDA-MB-231 and MCF-7 control and PA or DHA treated cells, Table S1: Fatty acid composition of MDA-MB-231 and MCF7 control and PA or DHA treated cells, Table S2: Endoplasmic reticulum fatty acid composition in MDA-MB-231 and MCF7 control and PA or DHA treated cells.

## References

[1] Fagnocchi L., Poli V., Zippo A. (2018). Enhancer reprogramming in tumor progression: A new route towards cancer cell plasticity. Cell. Mol. Life Sci..

[2] Pavlova N., Thompson C. (2016). The emerging hallmarks of cancer metabolism. Cell Metab..

[3] Zam W., Ahmed I., Yousef H. (2020). Warburg effects on cancer cells survival: The role of sugar starvation in cancer therapy. Curr. Clin. Pharmacol..

[4] Dennis E.A., Norris P.C. (2015). Eicosanoid storm in infection and inflammation. Nat. Rev. Immunol..

[5] Sulciner M.L., Gartung A., Gilligan M.M., Serhan C.N., Panigrahy D. (2018). Targeting lipid mediators in cancer biology. Cancer Metastasis Rev..

[6] Khadge S., Sharp J.G., Thiele G.M., McGuire T.R., Talmadge J.E. (2020). Fatty Acid Mediators in the Tumor Microenvironment. Adv. Exp. Med. Biol..

[7] Huang C., Freter C. (2015). Lipid metabolism, apoptosis and cancer therapy. Int. J. Mol. Sci..

[8] Currie E., Schulze A., Zechner R., Walther R.V., Farese T.C. (2013). Cellular Fatty Acid Metabolism and Cancer. Cell Metab..

[9] Nieman K.M., Romero I.L., Van Houten B., Lengyel N.B.E. (2013). Adipose tissue and adipocytes support tumorigenesis and metastasis. Biochim. Biophys. Acta.

[10] Qin C., Yang G., Yang J., Ren B., Wang H., Chen G., Zhao F., You L., Wang W., Zhao Y. (2020). Metabolism of pancreatic cancer: Paving the way to better anticancer strategies. Mol. Cancer..

[11] Curtis C., Shah S.P., Chin S.F., Turashvili G., Rueda O.M., Dunning M.J., Speed D., Lynch A.G., Samarajiwa S., Yuan Y. (2012). The genomic and transcriptomic architecture of 2,000 breast tumours reveals novel subgroups. Nature.

[12] Jung S.M., Kang D., Guallar E., Yu J., Lee J.E., Kim S.W., Nam S.J., Cho J., Lee S.K. (2020). Impact of Serum Lipid on Breast Cancer Recurrence. J. Clin. Med..

[13] Lowry O.H., Rosebrough N.J., Farr A.L., Randall R.J. (1951). Protein measurement with the Folin phenol reagent. J. Biol. Chem..

[14] Aoun M., Corsetto P.A., Nugue G., Montorfano G., Ciusani E., Crouzier D., Hogarth P., Gregory A., Hayflick S., Zorzi G. (2017). Changes in Red Blood Cell membrane lipid composition: A new perspective into the pathogenesis of PKAN. Mol. Genet. Metab..

[15] Ungaro F., Tacconi C., Massimin L., Corsetto P.A., Correale C., Fonteyne P., Piontini A., Garzarelli V., Calcaterra F., Della Bella S. (2017). MFSD2A Promotes Endothelial Generation of Inflammation-Resolving Lipid Mediators and Reduces Colitis in Mice. Gastroenterology.

[16] Corsetto P.A., Ferrara G., Buratta S., Urbanelli L., Montorfano G., Gambelunghe A., Chiaradia E., Magini A., Roderi P., Colombo I. (2016). Changes in Lipid Composition during Manganese-Induced Apoptosis in PC12 Cells. Neurochem. Res..

[17] Riaz M., van Jaarsveld M.T., Hollestelle A., Prager-van der Smissen W.J., Heine A.A., Boersma A.W., Liu J., Helmijr J., Ozturk B., Smid M. (2013). miRNA expression profiling of 51 human breast cancer cell lines reveals subtype and driver mutation-specific miRNAs. Breast Cancer Res..

[18] Sakamoto T., Niiya D., Seiki M. (2011). Targeting the Warburg effect that arises in tumor cells expressing membrane tyoe-1 matrix metalloproteinase. J. Biol. Chem..

[19] Pascual G., Avgustinova A., Mejetta S., Martín M., Castellanos A., Stephan-Otto Attolini C., Berenguer A., Prats N., Toll A., Hueto J.A. (2017). Targeting metastasis-initiating cells through the fatty acids receptor CD36. Nature.

[20] Corsetto P.A., Colombo I., Kopecka J., Rizzo A.M., Riganti C. (2017). ω-3 Long Chain Polyunsaturated Fatty Acids as Sensitizing Agents and Multidrug Resistance Revertants in Cancer Therapy. Int. J. Mol. Sci..

[21] Petan T., Jarc E., Jusović M. (2018). Lipid Droplets in Cancer: Guardians of Fat in a Stressful World. Molecules.

[22] Martin S., Parton R.G. (2006). Lipid droplets: A unified view of dynamic organelle. Nat. Rev. Mol. Cell. Biol..

[23] Freyre C.A., Rauher P.A., Ejsing C.S., Klemm R.W. (2019). MIGA2 Links Mitochondria, the ER, and Lipid Droplets and Promotes De Novo Lipogenesis in Adipocytes. Mol. Cell..

[24] Moon Y.A., Shah N.A., Mohapatra S., Warrington J.A., Horton J.D. (2001). Identification of a mammalian long chain fatty acyl elongase regulated by sterol regulatory element-binding proteins. J. Biol. Chem..

[25] Lai E., Bikopoulos G., Wheeler M.B., Rozakis-Adcock M., Volchuk A. (2008). Differential activation of ER stress and apoptosis in response to chronically elevated free fatty acids in pancreatic β-cells. Am. J. Physiol. Endocrinol. Metab..

[26] Harding S.V., Bateman K.P., Kennedy B.P., Rideout T.C., Jones P.J. (2015). Desaturationindex versus isotopically measured de novo lipogenesis as an indicator of acute systemic lipogenesis. BMC Res. Notes.

[27] Drąg J., Goździalska A., Knapik-Czajka M., Gawędzka A., Gawlik K., Jaśkiewicz J. (2017). Effect of high carbohydrate diet on elongase and desaturase activity and accompanying gene expression in rat’s liver. Genes Nutr..

[28] Schwartz L., Supuran C.T., Alfarouk K.O. (2017). The Warburg Effect and the Hallmarks of Cancer. Anticancer Agents Med. Chem..

[29] Chen J.Q., Russo J. (2012). Dysregulation of glucose transport, glycolysis, TCA cycle and glutaminolysis by oncogenes and tumor suppressors in cancer cells. Biochim. Biophys. Acta.

[30] Hensley C.T., Wasti A.T., DeBerardinis R.J. (2013). Glutamine and cancer: Cell biology, physiology, and clinical opportunities. J. Clin. Invest..

[31] Guo D., Bell E.H., Chakravarti A. (2013). Lipid metabolism emerges as a promising target for malignant glioma therapy. CNS Oncol..

[32] Menendez J.A., Lupu R. (2007). Fatty acid synthase and the lipogenic phenotype in cancer pathogenesis. Nat. Rev. Cancer..

[33] Guo D., Reinitz F., Youssef M., Hong C., Nathanson D., Akhavan D. (2011). An LXR agonist promotes glioblastoma cell death through inhibition of an EGFR/AKT/SREBP-1/LDLR-dependent pathway. Cancer Discov..

[34] Asghari A., Umetani M. (2020). Obesity and Cancer: 27-Hydroxycholesterol, the Missing Link. Int. J. Mol. Sci..

[35] Nelson E.R., Wardell S.E., Jasper J.F., Park S., Suchindran S., Howe M.K., Carver N.J., Pillai R.V., Sullivan P.M., Sondhi V. (2013). 27-Hydroxycholesterol Links Hypercholesterolemia and Breast Cancer Pathophysiology. Science.

[36] Sharma M., Tuaine J., McLaren B., Waters D.L., Black K., Jones L.M., McCormick S.P.A. (2016). Chemotherapy Agents Alter Plasma Lipids in Breast Cancer Patients and Show Differential Effects on Lipid Metabolism Genes in Liver Cells. PLoS ONE.

[37] Morada S.A.F., Cabota M.C. (2015). Tamoxifen regulation of sphingolipid metabolism—therapeutic implications. Biochim. Biophys. Acta.

[38] Kretzer I.F., Durvanei A.M., Guido M.G., Contente T.C., Maranhão R.C. (2016). Simvastatin increases the antineoplastic actions of paclitaxel carried in lipid nanoemulsions in melanoma-bearing mice. Int. J. Nanomed..

[39] Cho H.P., Nakamura M.T., Clarke S.D. (1999). Cloning, expression, and nutritional regulation of the mammalian delta-6 desaturase. J. Biol. Chem..

[40] Visca P., Sebastiani V., Botti C., Diodoro M.G., Lasagni R.P., Romagnoli F., Brenna A., De Joannon B.C., Donnorso R.P., Lombardi G. (2004). Fatty acid synthase (FA) is a marker of increased risk of recurrence in lung carcinoma. Anticancer Res..

[41] Huang J., Fan X.X., He J., Pan H., Li R.Z., Huang L., Jiang Z., Yao X.J., Liu L., Leung E.L. (2016). SCD1 is associated with tumor promotion, late stage and poor survival in lung adenocarcinoma. Oncotarget.

[42] Welte M.A., Gould A.P. (2017). Lipid droplet functions beyond energy storage. Biochim. Biophys. Acta.

[43] Walther T.C., Farese R.V. (2012). Lipid Droplets and Cellular Lipid Metabolism. Annu. Rev. Biochem..

[44] Dichlberger A., Schlager S., Maaninka K., Schneider W.J., Kovanen P.T. (2014). Adipose triglyceride lipase regulates eicosanoid production in activated human mast cells. J. Lipid Res..

[45] Schlager S., Goeritzer M., Jandl K., Frei R., Vujic N., Kolb D., Strohmaier H., Dorow J., Eichmann T.O., Rosenberger A. (2015). Adipose triglyceride lipase acts on neutrophil lipid droplets to regulate substrate availability for lipid mediator synthesis. J. Leukoc. Biol..

