# Supplementary material for: Exogenous Fatty Acids Modulate ER Lipid Composition and Metabolism in Breast Cancer Cells
Source: Cells. 2021 Jan 16;10(1):175. doi: 10.3390/cells10010175 (PMC7830208; doi:10.3390/cells10010175)
Supplement: Supplementary file 1 [file cells-10-00175-s001.pdf]

Exogenous Fatty Acids Modulate ER Composition and Lipid Metabolism in Breast Cancer Cells

Angela Maria Rizzo [1], Irma Colombo [1], Gigliola Montorfano [1], Stefania Zava [1] and Paola Antonia Corsetto [1,*]

Department of Pharmacological and Biomolecular Sciences, Università degli studi di Milano; angelamaria.rizzo@unimi.it; irma.colombo@unimi.it; gigliola.montorfano@unimi.it; stefania.zava@unimi.it
* Correspondence: paola.corsetto@unimi.it; Tel.: +39-0250315779

Supplemental Methods

2.1. Cell viability assay

The numbers of viable cells exposed to fatty acids (FA) were evaluated by the MTT (3-(4,5-dimethylthiazol-2-yl)-2,5-diphenyltetrazolium bromide) colorimetric assay. Initially, cells were seeded and cultured in 96-well plates for 48 h to allow adhesion to the plate and to reach 50-60% confluence. After this period, medium was replaced with fresh medium containing increasing concentrations (from 10 to 250 μM) of the experimental FA, that are palmitic acid (PA, C16:0) or docosahexaenoic acid (DHA, C22:6). The final concentration of ethanol (<1%) in the culture medium had no antiproliferative effect on any cell line tested. Cells were grown for 72 h and then 10 μl of MTT stock solution (5 mg/ml in PBS, pH 7.5) were added to each well. After 4 h of incubation, and addition of 100 μl of solubilizing solution (10% SDS in 0.01M HCl) was added, cells were incubated overnight. Plates were read at 540 nm in a TECAN plate reader. Data points represent the mean of eight wells of three independent experiments and the results are expressed as relative growth rate (RGR) in comparison to controls that were exposed to a concentration of ethanol equal to that in the samples exposed to fatty acids.

2.2. Annexin V and Dead Cell Muse Analysis

The apoptotic process was assayed by using of the Muse™ Annexin V & Dead Cell kit, following manufacturer's instructions.

For cytofluorimetric analysis, cells were seeded on 6-well at a specific cell density: $1.5x10^4$ cells/cm^2 for MDA-MB-231 cells and $3.0x10^4$ cells/cm^2 for MCF7 cells. After 48 h from seeding the cells were exposed to 50 μM of palmitic acid (PA, C16:0) or docosahexaenoic acid (DHA, C22:6). After 72 h of treatment, the cells were trypsinized and centrifuged at 1000 g for 5 minutes at 24°C.

The cell pellets were then dissolved in DMEM medium with 10% v / v FBS. 100 μL of the reagent from the "Muse Annexin V & Dead Cell Kit" was added to the control and treated samples, incubated for 20 minutes at dark and room temperature. Finally, the samples were read by Muse Cell Analyzer to quantitate live, early and late apoptosis, and dead cells.

Supplemental Figures

MDA-MB-231 **MCF7**

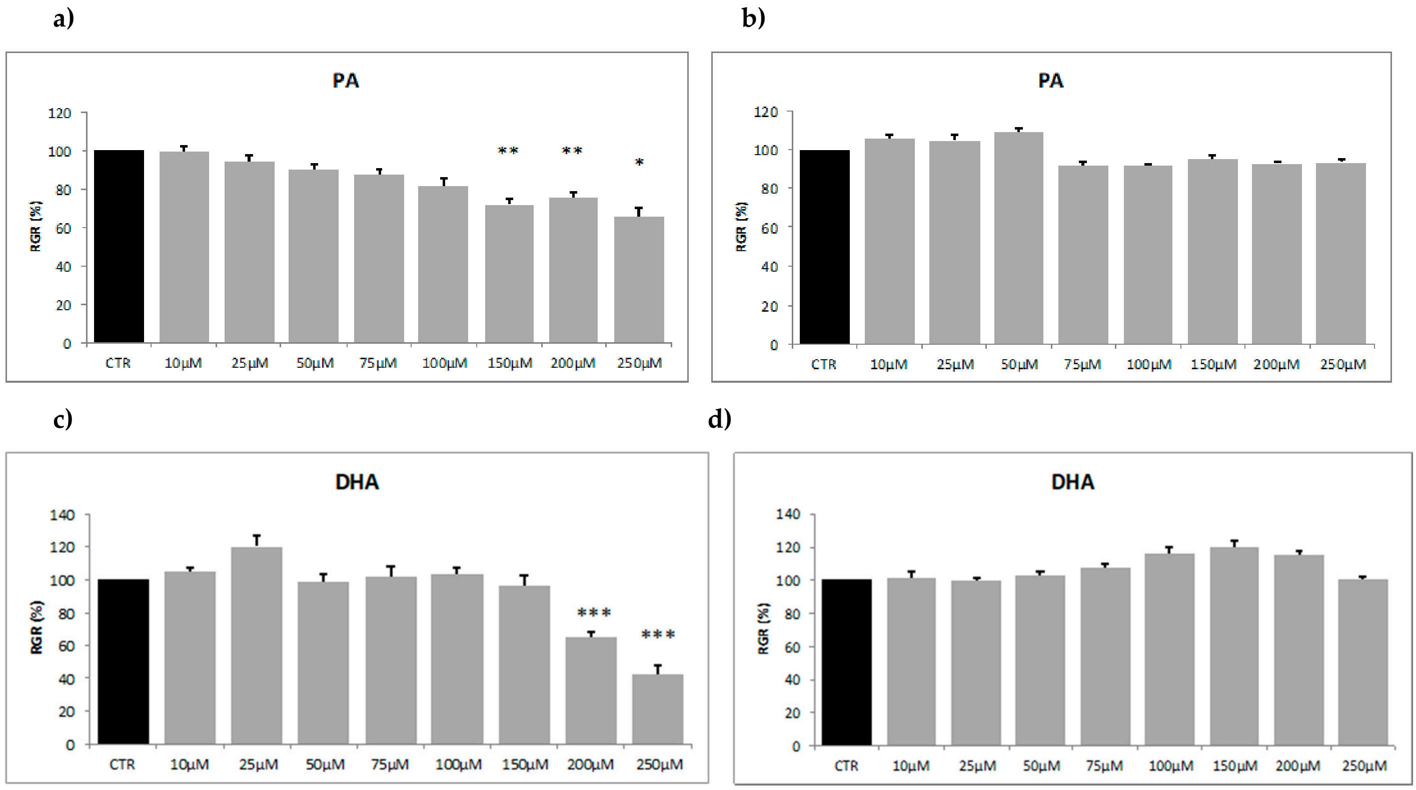

Figure S1: Effects of palmitic acid (PA) and docosahexaenoic acid (DHA) on cell viability of breast cancer cells MDA-MB-231 (panels a and c) and MCF7 (panels b and d) analyzed by MTT assay. Cells are treated with increasing concentrations of PA and DHA for 72 h. Data are expressed as Relative Growth Rate (RGR) in comparison with controls and represented as mean ± SE of three independent experiments. * $p < 0.05$; ** $p < 0.01$; ***$p < 0.01$.

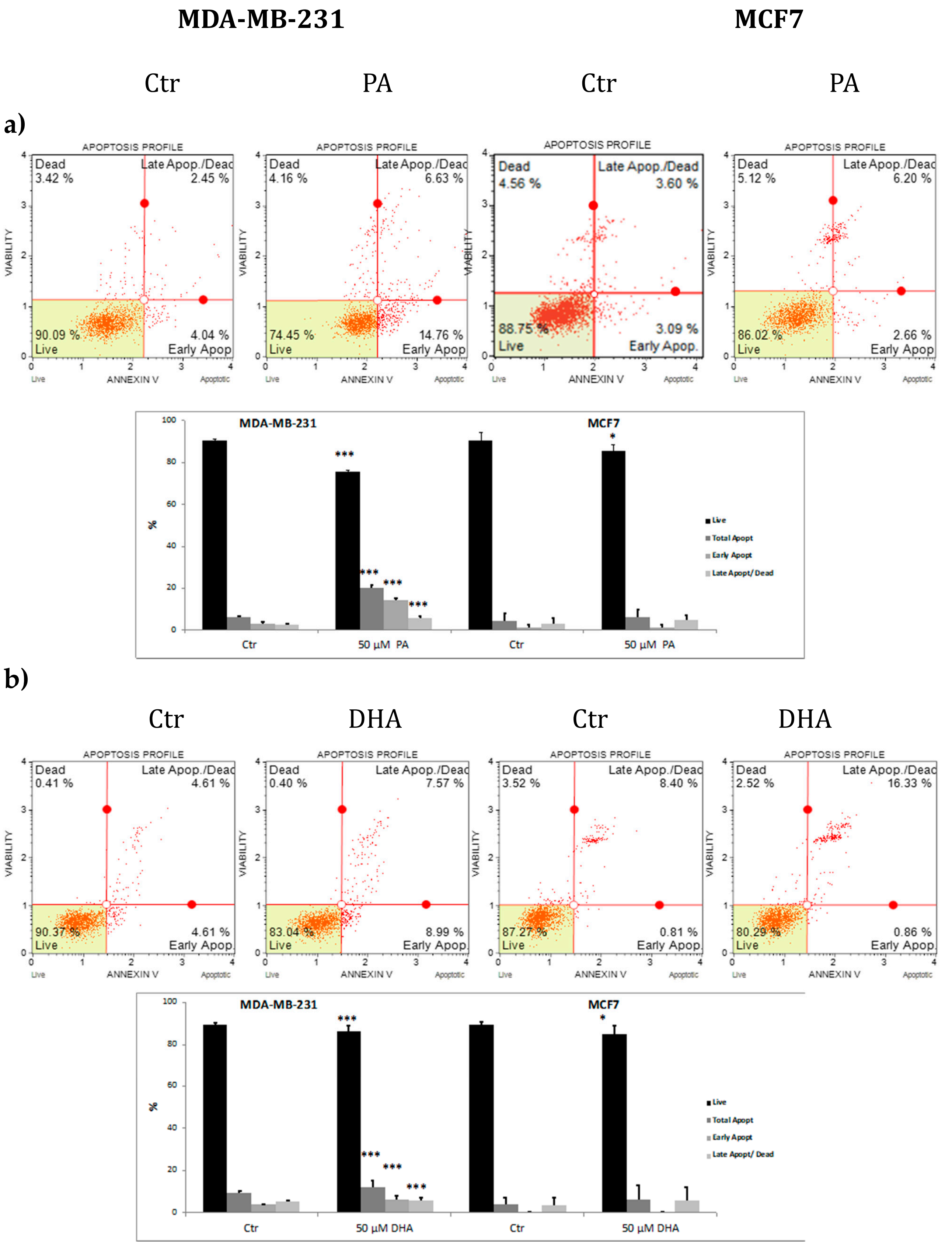

Figure S2: Analysis of the apoptotic process by Annexin V cytofluorimetry in MDA-MB-231 and MCF-7 control (Ctr) and treated cells with 50 µM palmitic acid (PA) (panel a) or 50 µM docosahexaenoic acid (DHA) (panel b) for 72 h. Data are represented as means ± SE obtained from three independent experiments. *$p<0.05$, ***$p<0.001$ vs Ctr cells of the same line.

Supplemental Tables

Table S1: Fatty acid composition of MDA-MB-231 and MCF-7 breast cancer cells, before and after treatment with 50 μM PA or DHA.

	MDA-MB-231			MCF7		
	Ctr	PA	DHA	Ctr	PA	DHA
C16:0	20.1±0.218	27.9±0.670***	17.9±0.414***	21.0±0.367§	20.9±0.373	20.7±0.241
C16:1	3.51±0.110	3.64±0.265	2.98±0.230	10.2 ±0.390§§§	10.2±0.336	7.48±0.846*
C18:0	19.1±0.163	18.72±0.404	15.3±0.579***	17.0 ±0.335§§§	17.5±0.435	18.5±0.680
C18:1	30.0±0.226	24.1±0.615***	22.1 ±0.904***	30.3 ±0.317	29.9±0.536	22.9±1.03***
C18:2	4.97±0.135	4.07±0.129***	4.73±0.327	3.68±0.110§§§	3.64±0.123	3.14±0.084***
C18:3 n-6	0.549±0.068	0.856±0.250	0.872±0.275	0.498±0.119	0.503±0.091	0.429±0.093
C18:3 n-3	1.57±0.056	1.73±0.278	1.94±0.477	1.51±0.121	1.49±0.184	1.04±0.118*
C20:3	1.16±0.041	1.20±0.054	1.06±0.049	1.56±0.056§§§	1.59±0.038	1.12±0.041***
C20:4	10.6±0.170	9.033±0.337***	6.63±0.387***	9.44±0.250§§§	9.69±0.360	6.96±0.237***
C20:5	0.823±0.082	2.01±0.800	1.45±0.061***	1.52±0.201§§	1.29±0.163	3.87±0.391***
C22:5	3.20±0.130	2.99±0.215	3.01±0.114	0.407±0.015§§§	0.506±0.093	0.624±0.027***
C22:6	4.30±0.077	3.82±0.071***	22.1±1.04***	2.77±0.089§§§	2.87±0.107	13.3±1.483***
SFA	39.3±0.204	46.6±0.856***	33.2±0.787***	38.1±0.622	38.4±0.640	39.2 ±0.663
MUFA	33.5±0.214	27.7±0.652***	25.1 ±0.863***	40.5±0.665§§§	40.0±0.732	30.4±1.85***
PUFA	27.2±0.303	25.7±1.12	41.7 ±1.59***	21.4±0.190§§§	21.6±0.388	30.4±1.42***
n-6 PUFA	17.3±0.229	15.2±0.349***	13.3±0.403***	15.2±0.297§§§	15.4±0.454	11.6±0.410***
n-3 PUFA	9.89±0.212	10.6±0.847	28.5±1.563***	6.21±0.213§§§	6.16±0.257	18.8±1.75***

Data are expressed as percentage of total fatty acids (mean ± SE of four independent experiments) *p<0.05, ***p<0.001 vs Ctr cells of the same line; § p<0.05, §§ p<0.01, §§§ p<0.001 vs MDA-MB-231 Ctr cells. SFA, saturated fatty acids (=sum of saturated fatty acids); MUFA, monounsaturated fatty acids (=sum of monounsaturated fatty acids); PUFA, polyunsaturated fatty acids (=sum of n-6 and n-3 PUFA); n-6 PUFA, omega-6 polyunsaturated fatty acids; n-3 PUFA, omega-3 polyunsaturated fatty acids.

Table S2: ER fatty acid composition of MDA-MB-231 and MCF-7 breast cancer cells, before and after treatment with 50 μM PA or DHA

	MDA-MB-231			MCF7		
	Ctr	PA	DHA	Ctr	PA	DHA
C16:0	20.7±0.791	25.3±1.412*	18.7±0.400	21.5±0.231	21.1±0.874	20.9±0.274
C16:1	4.87±0.588	4.43±1.38	3.62±0.364	10.1±0.448§§§	10.1±0.810	8.08±1.15
C18:0	17.4±0.595	19.1±1.48	16.6±0.783	17.5±0.473	18.6±0.777	19.2±1.07
C18:1	29.6±0.576	26.0±1.15*	22.7±0.735***	28.4±0.355	27.6±0.459	20.6±0.464***
C18:2	4.82±0.352	4.30±0.326	6.46±2.39	3.51±0.153§§	3.42±0.145	3.38±0.262
C18:3 n-6	0.430±0.135	0.686±0.366	0.433±0.164	0.429±0.126	0.567±0.296	0.595±0.382
C18:3 n-3	1.52±0.172	0.938±0.267	0.749±0.233*	1.20±0.043	1.18±0.072	0.992±0.061*
C20:3	1.09±0.060	1.06±0.094	1.02±0.046	1.53±0.051§§§	1.54±0.085	1.04±0.156*
C20:4	12.3±0.868	12.0±0.846	9.24±0.214**	10.7±0.297	10.7±0.097	8.92±0.296**
C20:5	0.903±0.128	0.701±0.063	1.35±0.454	1.55±0.093§§	1.73±0.158	5.10±0.181***
C22:5	2.56±0.160	2.02±0.245	2.19±0.099	0.457±0.044§§§	0.410±0.021	0.633±0.087
C22:6	3.81±0.259	3.40±0.287	17.0±1.46**	3.16±0.090§	3.11±0.191	10.6±0.500**
SFA	38.1±0.765	44.4±0.595***	35.3±1.05	39.0±0.390	39.7±0.865	40.1±1.150
MUFA	34.5±0.768	30.4±0.884**	26.3±0.776**	38.5 ±0.662§§	37.7±1.235	28.7±1.339**
PUFA	27.41±1.41	25.1±0.863	38.4±1.61	22.5±0.450§§	22.6±0.537	31.2±0.332***
n-6 PUFA	18.6±1.09	18.1±0.807	17.2±2.32	16.1±0.412	16.2±0.298	13.9±0.440**
n-3 PUFA	8.79±0.455	7.06±0.418*	21.2±1.92*	6.37±0.138§§§	6.43±0.310	17.3±0.464***

Data are expressed as percentage of total fatty acids (mean ± SE of four independent experiments) *p<0.05, **p<0.01,***p<0.001 vs Ctr cells of the same line; §§ p<0.01, §§§ p<0.001 vs MDA-MB-231 Ctr cells. SFA, saturated fatty acids (=sum of saturated fatty acids); MUFA, monounsaturated fatty acids (=sum of monounsaturated fatty acids); PUFA, polyunsaturated fatty acids (=sum of n-6 and n-3 PUFA); n-6 PUFA, omega-6 polyunsaturated fatty acids; n-3 PUFA, omega-3 polyunsaturated fatty acids.
